# Supplementary material for: Complex I is bypassed during high intensity exercise
Source: Nat Commun. 2019 Nov 7;10:5072. doi: 10.1038/s41467-019-12934-8 (PMC6838197; doi:10.1038/s41467-019-12934-8)
Supplement: Supplementary file 1 — Supplementary Information [file 41467_2019_12934_MOESM1_ESM.pdf]

Supplementary Information

Complex I is bypassed during high intensity exercise

Nilsson et al.

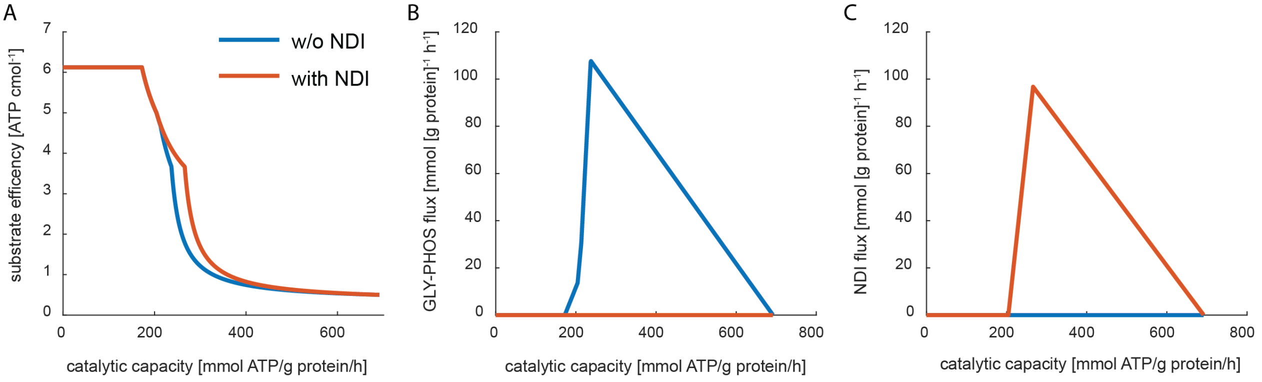

Figure 1 Substrate efficiency with and without complex I bypass enzyme from yeast (NDI). A) Addition of the yeast NDI reaction to the model increased the attainable area in pareto plot of catalytic capacity vs substrate efficiency. The NDI enzyme was assumed to have a specific activity of 500 mmol mg^{-1} min^{-1}. B) The predicted flux through the GLY-PHOS shuttle was abolished in the presence of the NDI enzyme. C) the NDI reaction carried similar flux as GLY-PHOS reaction did in the absence of NDI.

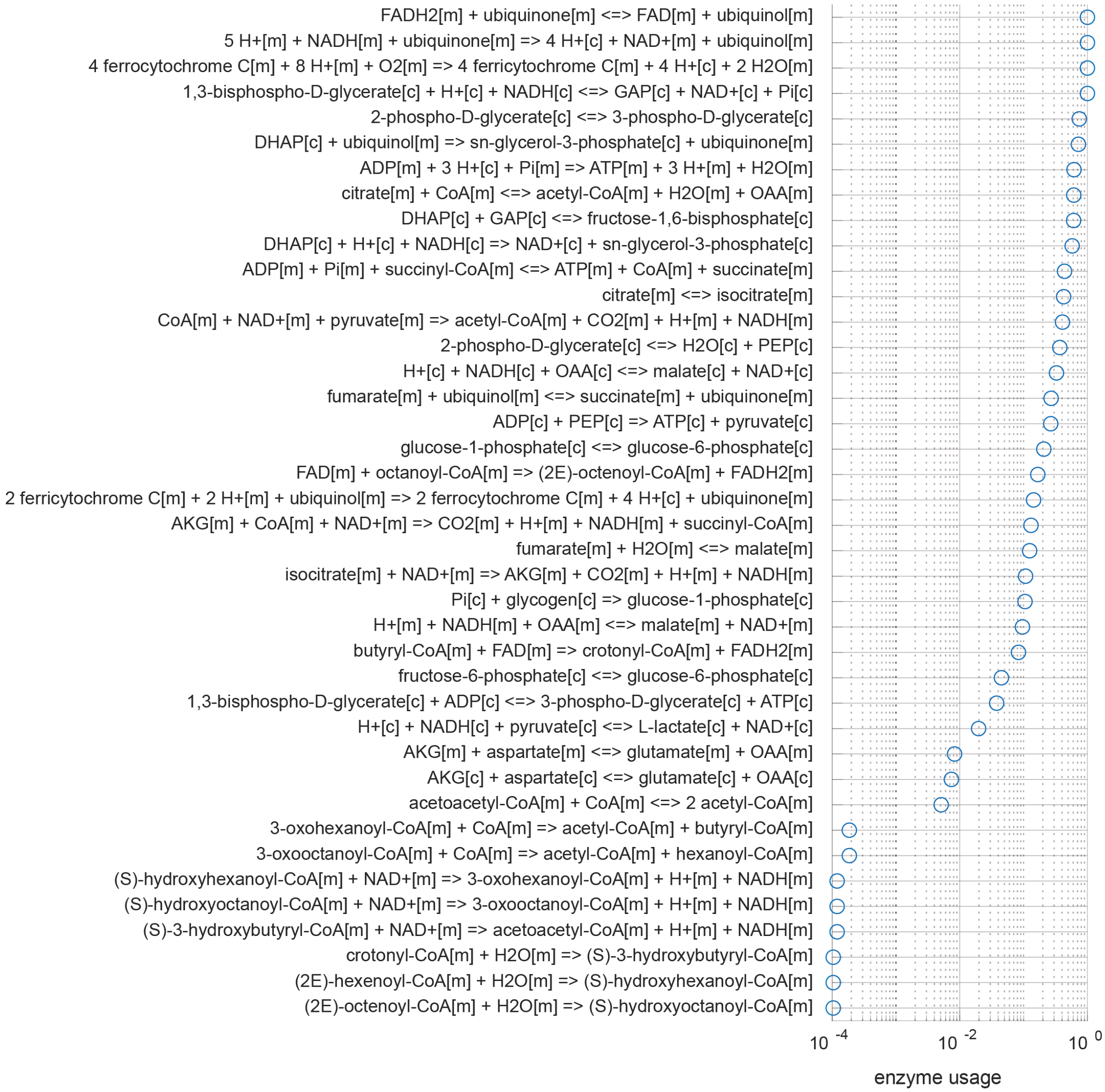

Figure 2 The effective enzyme usage for each reaction. For each reaction the effective v_{max} is compared with the maximum absolute flux amongst all investigated ATP production intensities as [max flux] per [effective v_{max}].

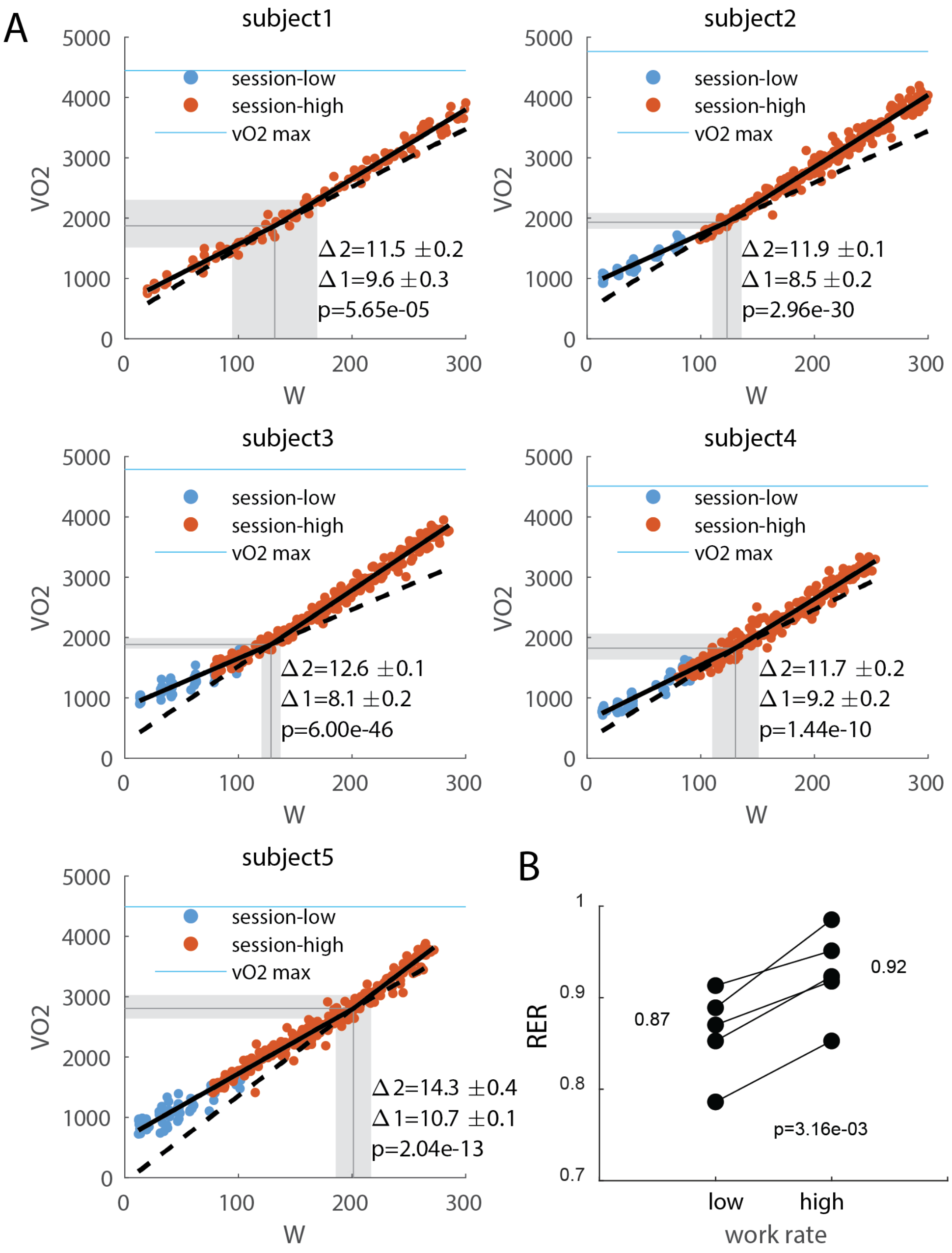

Figure 3 Fitted slopes below and above the inflection point for 5 subjects. A) Non-linear least squares fit of the inflection point in vO2, and its 95% confidence interval was calculated using the lsqcurvefit function in matlab. The slope before and after the inflection point (± s.e.) was calculated using a linear regression model and the fitlm function in matlab. The significance of the difference in slope was given as the t-statistic of the additional slope coefficient (H) for a linear model vO2=W:(1+H) for work rates (W) centered to the inflection point. For subjects 2-5 the exercise was divided in two sessions with a short break in-between to accommodate requirements for comfort. We are aware that the slope has the unit ml[min]$^{-1}$J^{-1}s^{-1}, which could be further simplified, however, these are preferred units by convention. B) Median RER at low and high work rates. Statistics calculated with a paired two tailed t-test, tested for normality using the Lilliefors test.

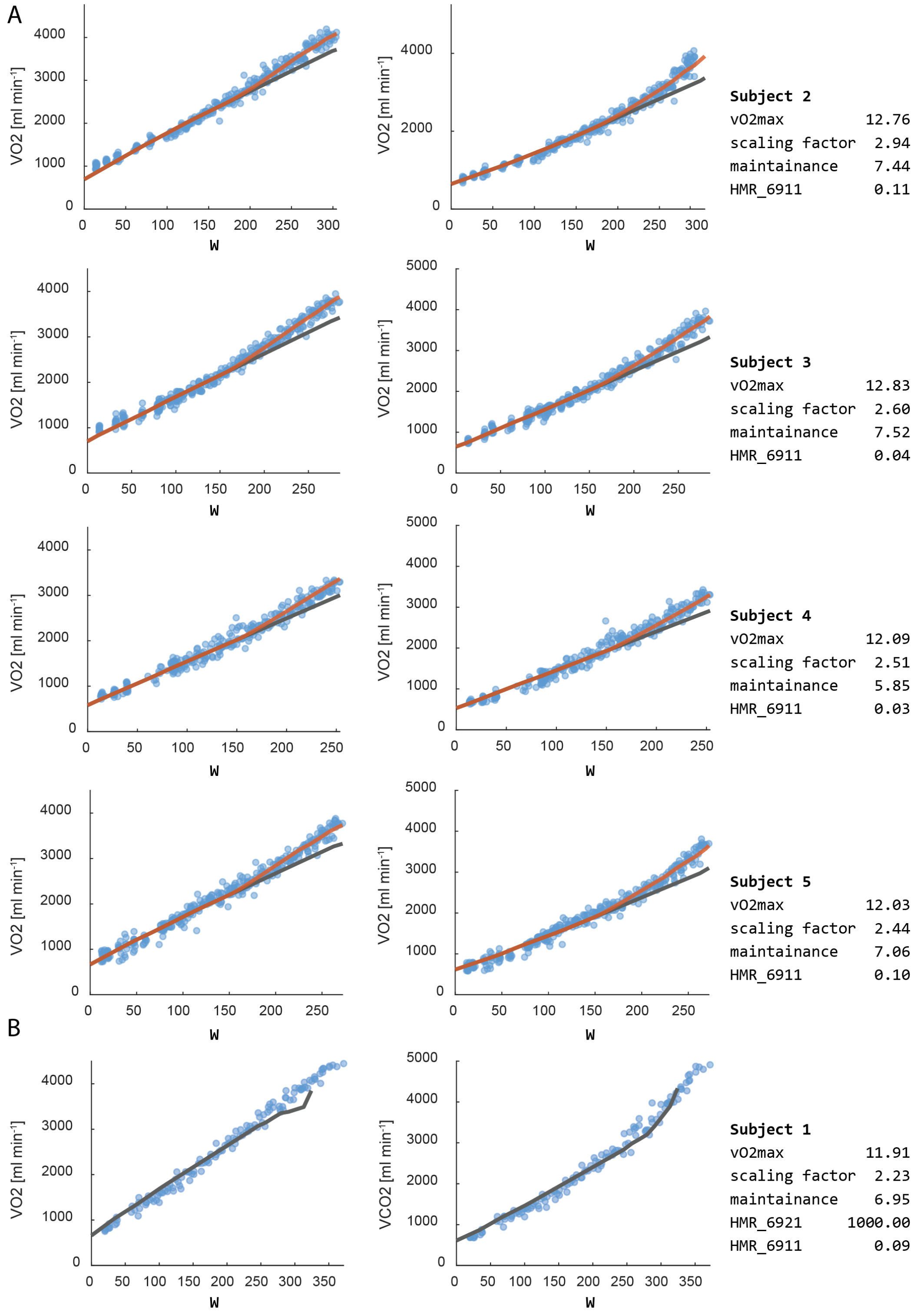

Figure 4 Fitted models for multiple subjects. A) best fit model (orange lines) for 4 subjects optimizing the parameters maintenance, scaling factor and fat oxidizing capacity (HMR_6911), the value for vO_2 max was taken directly from the VO2 max test. The curve in absence of complex I bypass (grey line) shown for reference. B) A comparably poor fit was attained when the complex I capacity parameter (HMR_6921) was left unconstrained.

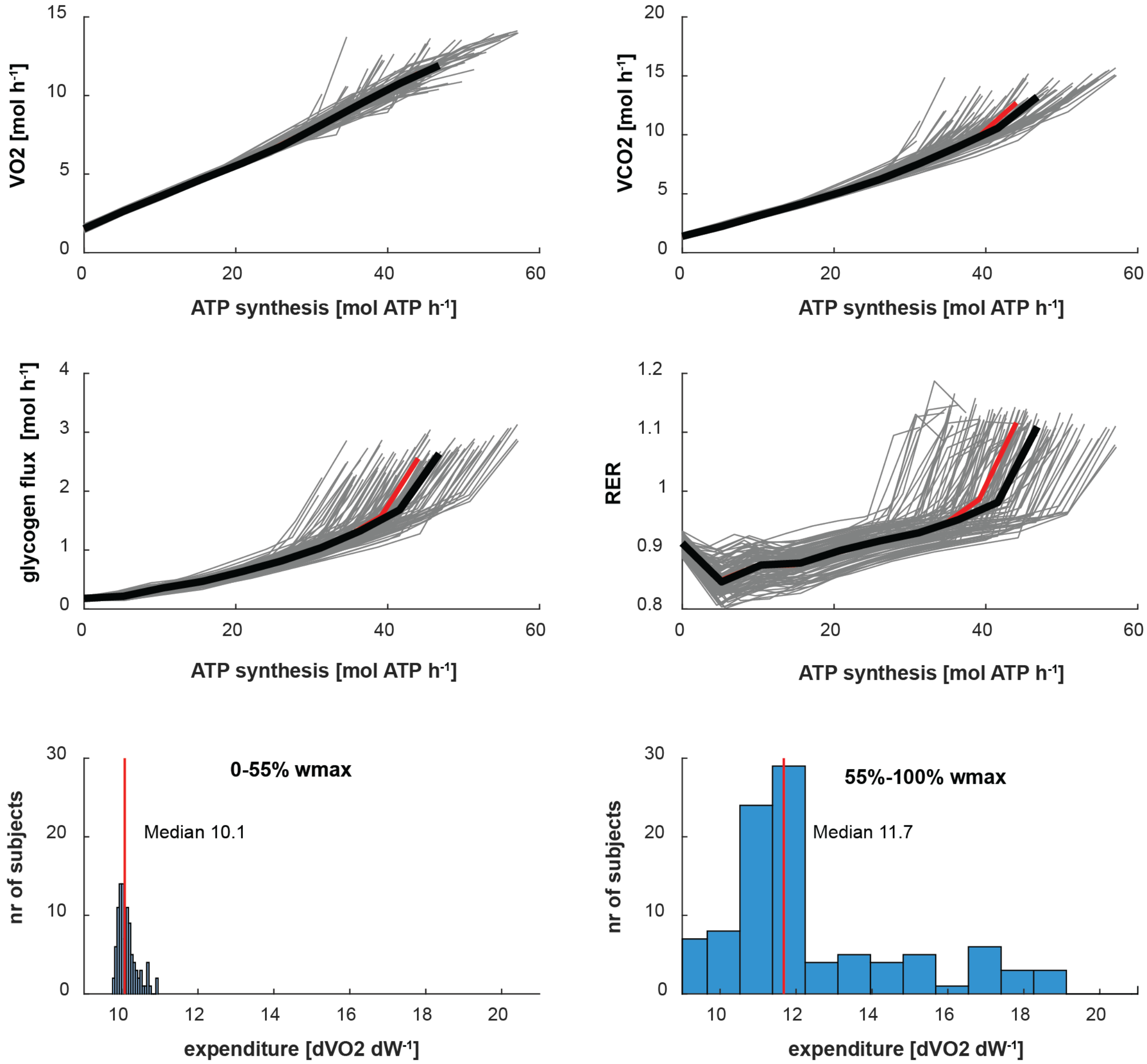

Figure 5 Sensitivity analysis of the model. All parameters of the multi-tissue model were perturbed by a random value uniformly sampled on the interval +-20% of the initial value. The result of 100 simulations shown above. The black curve shows the reference condition, the gray curves are individual perturbed simulations, and the red curve shows the average of all simulations. Histograms of dvO2 per dW at low (0-55% of wmax) and high (55-100% wmax) work rates.

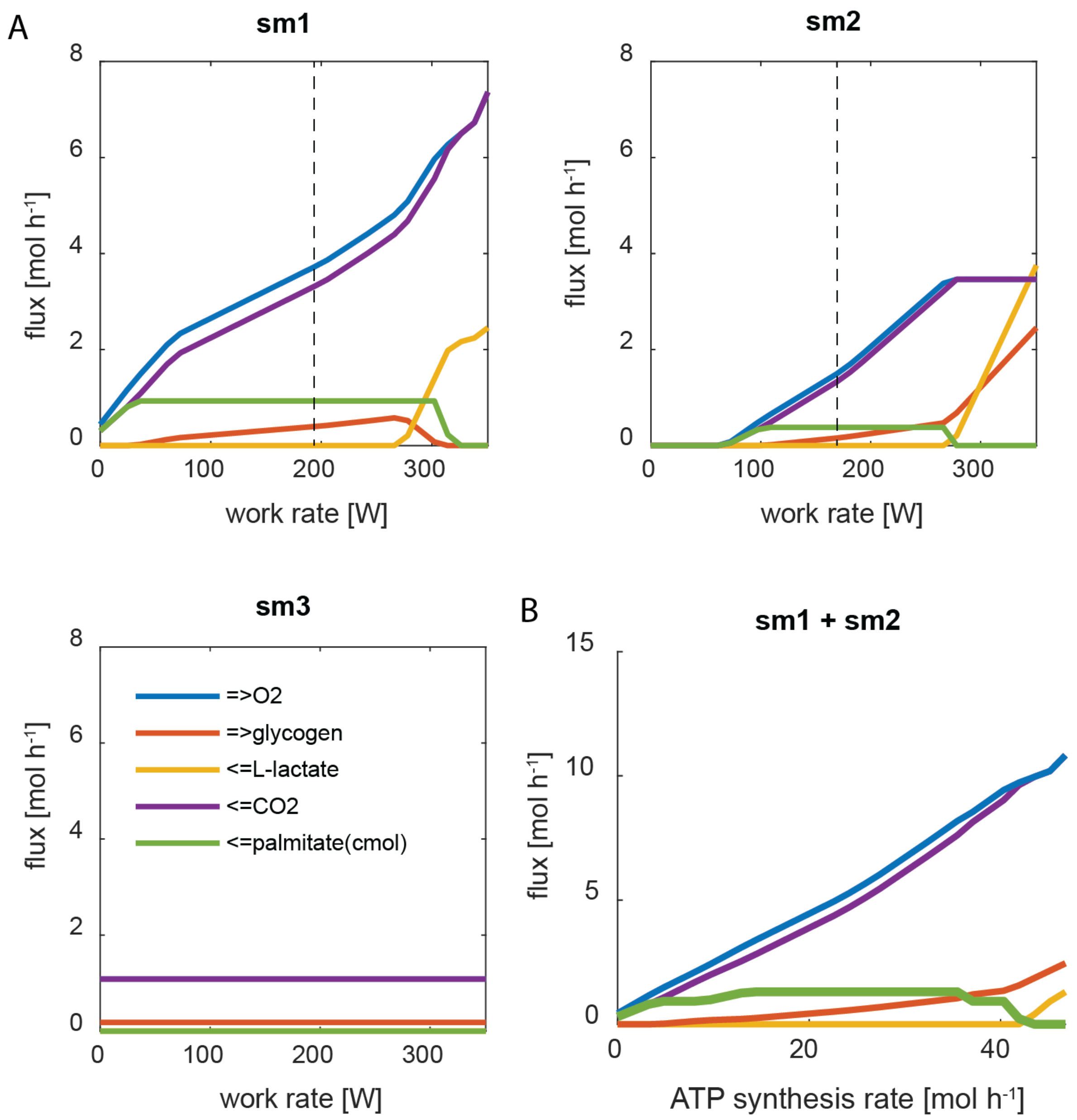

Figure 6 Metabolic fluxes between the sub-models. A) The intracellular consumption (=>) and production fluxes (<=) in the three different tissues. The model shows a flux of lactate from m2 towards m1 after complex IV is saturated in m2. The bypass of complex I in each fiber type is indicated with a dashed line. B) The net-fluxes over the whole muscle attained by adding sm1 and sm2.

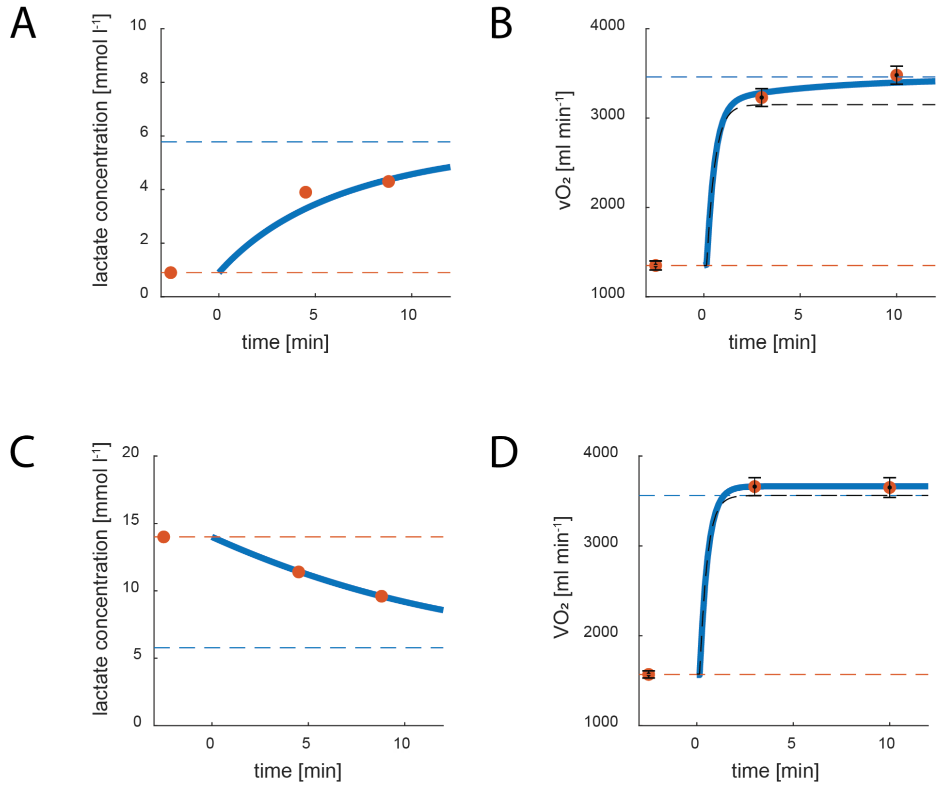

Figure 7 Dynamic model of lactate concentration and oxygen flux at high work rates, with and without prior exercise. An ordinary differential equation system was set up with a basal lactate flux of 0.5 mol per h and an additional lactate flux of 1.8 mmol per h from the exercising muscle. The lactate filled a reservoir with an estimated volume of 37 L, corresponding to blood and muscle tissue. The lactate efflux from the reservoir was model as a Michaelis–Menten equation with a kM for lactate of 10.73 mM[1] and a manually fitted v_{max} of 6.6 mol per h. The oxygen flux in absence of lactate was estimated by an exponential equation from literature[2] as **d**ynamic models have previously shown that the oxygen dynamics during the first few minutes of exercise can be derived from transient changes in phosphocreatine[3]. The oxygen flux in the presence of lactate was calculated by the fraction of oxygen flux derived from lactate and an assumed increase in oxygen expenditure when using lactate as carbon source of 16%, based on the increased oxygen expenditure at rest in the presence of lactate, 1.57 vs 1.35 L per min[2] (pseudo code in Supplementary Methods). A) Simulations (blue lines) show that lactate accumulates in blood and tissues before reaching steady state (blue dashed line) for subjects without prior exercise, in agreement with measurements from literature from n=8 subjects (orange dots)[2], error bars show s.e.m. The baseline (orange dashed line) is taken a few minutes before the exercises. B) After the initial adjustment given by an empirical formula (black dashed line), there was a slow upward drift in oxygen flux driven by the increased usage of lactate as carbon source, which has lower oxygen efficiency than glycogen. C) Lactate concentrations decreased during the exercise when initial levels were higher than the steady state values due to prior exercise. D) High lactate concentrations increased the oxygen consumption rate, but there was no upward drift since lactate uptake was at full capacity under these conditions.

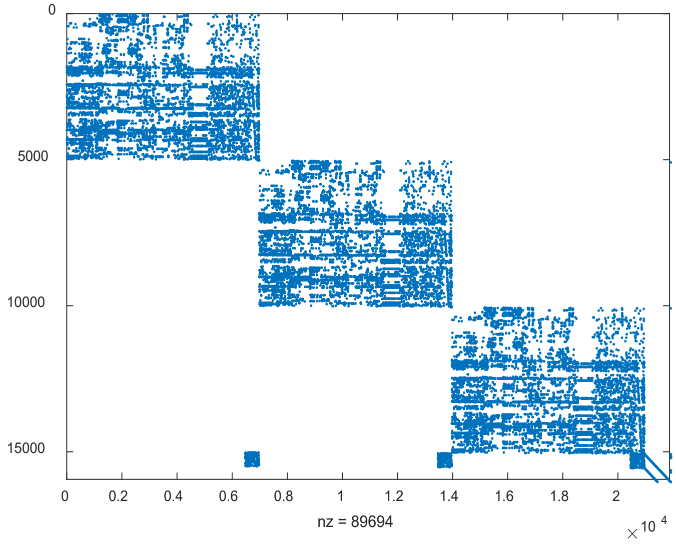

Figure 7 Structure of the stochiometric matrix for the multi tissue model. Three GEMs, m1, m2 and m3 (in order from top left) are joined by a compartment (bottom). Exchange reactions are introduced between this compartment and the boundary (bottom right).

Table 1 Table of specific activity (SA) parameters (µmol $[min]^{-1}$[mg purified protein]$^{-1}$) used in the enzyme constrained model.

Reaction	Equation	EC	SA	Organism	Ref.
**Glycolysis Gluconeogenesis**					
HMR_4388	NADH + pyruvate <=> L-lactate + NAD+	1.1.1.27	4034	Human	Brenda
HMR_4379	ATP + fructose-6-phosphate => ADP + fructose-1,6-bisphosphate	2.7.1.11	99.5	Human	Brenda
HMR_4358	ADP + PEP => ATP + pyruvate	2.7.1.40	420	Human	Brenda
HMR_4363	2-phospho-D-glycerate <=> H2O + PEP	4.2.1.11	75	Human	Brenda
HMR_4365	2-phospho-D-glycerate <=> 3-phospho-D-glycerate	5.4.2.11	515	Human	Brenda
HMR_4368	1,3-bisphospho-D-glycerate + ADP <=> 3-phospho-D-glycerate + ATP	2.7.2.3	2260	Human	Brenda
HMR_4373	1,3-bisphospho-D-glycerate + NADH <=> GAP + NAD+ + Pi	1.2.1.12	620	Human	Brenda
HMR_4375	DHAP + GAP <=> fructose-1,6-bisphosphate	4.1.2.13	22.3	Human	Brenda
HMR_4381	fructose-6-phosphate <=> glucose-6-phosphate	5.3.1.9	870	Human	Brenda
HMR_4391	DHAP <=> GAP	5.3.1.1	10240	Human	Brenda
HMR_4396	glucose-1-phosphate <=> glucose-6-phosphate	5.4.2.2	1442	Human	Brenda
**Tricarboxylic acid cycle**					
HMR_4137	CoA+ NAD++ pyruvate=> acetyl-CoA + CO2 + NADH[m]	1.2.4.1	26	Human	Brenda
HMR_3957	isocitrate+ NAD+=> AKG+ CO2 + NADH[m]	1.1.1.41	29	Human	Brenda
HMR_4139<br>HMR_4141	NADH + OAA <=> malate + NAD+	1.1.1.37	47	Human	Brenda
HMR_4145	citrate+ CoA<=> acetyl-CoA+ H2O+ OAA[m]	2.3.3.1	194	Pig	Brenda*
HMR_4152	ADP+ Pi+ succinyl-CoA<=> ATP+ CoA+ succinate[m]	6.2.1.5	110	Human	[4]
HMR_4410	fumarate+ H2O<=> malate[m]	4.2.1.2	450	Pig	Brenda
HMR_4456	citrate<=> isocitrate[m]	4.2.1.3	21.4	Bovine	Brenda
HMR_4652	fumarate+ ubiquinol<=> succinate+ ubiquinone[m]	1.3.5.1	78	"Mamalia"	Brenda
HMR_5297	AKG+ CoA+ NAD+=> CO2+ NADH+ succinyl-CoA[m]	1.2.4.2	18.5	Bovine	[5]
**Oxidative phosphorylation**					
HMR_6914	4 ferrocytochrome C + 8 H+ + O2 => 4 ferricytochrome C + 4 H+ + 2 H2O	1.9.3.1	24	Human	**
HMR_6916	ADP + 3 H+ + Pi => ATP + 3 H+ + H2O	3.6.3.14	11	Human	***
HMR_6918	2 ferricytochrome C + 2 H+ + ubiquinol => 2 ferrocytochrome C + 4 H+ + ubiquinone	1.10.2.2	160	Bovine	Brenda
HMR_6921	5 H+ + NADH + ubiquinone => 4 H+ + NAD+ + ubiquinol	1.6.5.3	6.6	Sheep	[6]
HMR_6911	FADH2 + ubiquinone <=> FAD + ubiquinol	1.5.5.1	17.6	Human	Brenda
**Other**					
HMR_3827<br>HMR_3829	AKG + aspartate <=> glutamate + OAA	2.6.1.1	170	Pig	Brenda
HMR_0479<br>HMR_0483	DHAP + NADH => NAD+ + sn-glycerol-3-phosphate	1.1.1.8	250	Human	Brenda
GP	Pi[c] + glycogen[c] => glucose-1-phosphate[c]	2.4.1.1	30	Bovine	Brenda

*Calculated from a turnover value of 167 s-1 and a mass of 51.6 kDa [7].**) Value calculated from a turnover value of 80 s^{-1} [8] and a mass of 200 [9]. ***) Calculated from a turnover value of 110 s^{-1} [10] and a mass of 600 kDa [9].

For values with reference to Brenda [11] the highest value for human was selected. If no value existed for human, values from other mammals were used as indicated. If there were no value available in Brenda a literature search was performed.

Table 2 Table of SA parameters for beta oxidation of fatty acids.

Reaction	Equation	EC	SA	Organism	Ref.
HMR_0174	AMP + octanoyl-CoA + PPi <=> ATP + CoA + octanoic acid	6.2.1.2	4.15	Mouse	Brenda
HMR_0217	AMP + palmitoyl-CoA + PPi <=> ATP + CoA + palmitate	6.2.1.3	28.7	Rat	Brenda
HMR_3149<br>HMR_3163<br>HMR_3121<br>HMR_3128<br>HMR_3135<br>HMR_3142	FAD + octanoyl-CoA => (2E)-octenoyl-CoA + FADH2<br>FAD + butyryl-CoA => crotonyl-CoA + FADH2<br>FAD + palmitoyl-CoA => (2E)-hexadecenoyl-CoA + FADH2<br>FAD + myristoyl-CoA => (2E)-tetradecenoyl-CoA + FADH2<br>FAD + lauroyl-CoA => (2E)-dodecenoyl-CoA + FADH2<br>FAD + decanoyl-CoA => (2E)-decenoyl-CoA + FADH2	1.3.8.7	24.9	Human	Brenda
HMR_3156	FAD + hexanoyl-CoA => (2E)-hexenoyl-CoA + FADH2	1.3.8.1	7.4	Human	Brenda
HMR_3150<br>HMR_3157<br>HMR_3164<br>HMR_3122<br>HMR_3129<br>HMR_3136<br>HMR_3143	(2E)-octenoyl-CoA + H2O => (S)-hydroxyoctanoyl-CoA<br>(2E)-hexenoyl-CoA + H2O => (S)-hydroxyhexanoyl-CoA<br>crotonyl-CoA + H2O => (S)-3-hydroxybutyryl-CoA<br>(2E)-hexadecenoyl-CoA + H2O => (S)-3-hydroxyhexadecanoyl-CoA<br>(2E)-tetradecenoyl-CoA + H2O => (S)-3-hydroxytetradecanoyl-CoA<br>(2E)-dodecenoyl-CoA + H2O => (S)-3-hydroxydodecanoyl-CoA<br>(2E)-decenoyl-CoA + H2O => (S)-hydroxydecanoyl-CoA	4.2.1.17	1334	Pig	Brenda
HMR_3151<br>HMR_3158<br>HMR_3166<br>HMR_3123<br>HMR_3130<br>HMR_3137<br>HMR_3144	(S)-hydroxyoctanoyl-CoA + NAD+ => 3-oxooctanoyl-CoA + NADH<br>(S)-hydroxyhexanoyl-CoA + NAD+ => 3-oxohexanoyl-CoA + NADH<br>(S)-3-hydroxybutyryl-CoA + NAD+ => acetoacetyl-CoA + NADH<br>(S)-3-hydroxyhexadecanoyl-CoA + NAD+ => 3-oxopalmitoyl-CoA + NADH<br>(S)-3-hydroxytetradecanoyl-CoA + NAD+ => 3-oxotetradecanoyl-CoA + NADH<br>(S)-3-hydroxydodecanoyl-CoA + NAD+ => 3-oxododecanoyl-CoA + NADH<br>(S)-hydroxydecanoyl-CoA + NAD+ => 3-oxodecanoyl-CoA + NADH	1.1.1.35	1200	Human	Brenda
HMR_3153<br>HMR_3160<br>HMR_3125<br>HMR_3132<br>HMR_3139<br>HMR_3146	3-oxooctanoyl-CoA + CoA => acetyl-CoA + hexanoyl-CoA<br>3-oxohexanoyl-CoA + CoA => acetyl-CoA + butyryl-CoA<br>3-oxopalmitoyl-CoA + CoA => acetyl-CoA + myristoyl-CoA<br>3-oxotetradecanoyl-CoA + CoA => acetyl-CoA + lauroyl-CoA<br>3-oxododecanoyl-CoA + CoA => acetyl-CoA + decanoyl-CoA<br>3-oxodecanoyl-CoA + CoA => acetyl-CoA + octanoyl-CoA	2.3.1.16	597	Pig	Brenda
HMR_3885	acetoacetyl-CoA + CoA <=> 2 acetyl-CoA	2.3.1.9	130	Rat	Brenda
HMR_2626	CoA + L-palmitoylcarnitine <=> L-carnitine + palmitoyl-CoA	2.3.1.21	47.5	Bovine	Brenda
HMR_2630	CoA + L-palmitoylcarnitine <=> L-carnitine + palmitoyl-CoA (mitochondrial)	2.3.1.7	240	Rat	Brenda

Table 3 SA values for Complex I found in literature.

Organism	SA	Reference
Ovis aries	6.6	[12]
Bos Taurus	3.7	[13]
Bos Taurus	2.42	[14]
Yarrowia lipolytica	4.3	[15]
Yarrowia lipolytica	4.2	[16]
**Mean**	4.2±1.5	

In literature there are also turnover rates that have been estimated in sub mitochondrial particles from assumed protein concentrations. These rates can be recalculated to specific activities using a molecular mass of 980 kDa [9], but they were not included in the analysis since complex I had not been isolated. The rates are 250 s^{-1} (15.3 U per mg) [17], 211.54 s^{-1} (12.9 U per mg) [18], 166 s^{-1} (10.2 U per mg) [19] and 50 s^{-1} (3.1 U per mg) [20].

Table 4 Parameters used in the multi tissue model.

Parameter	Unit	Value	Reference
dwMuscle	kg	20·0.2	Estimated
muscleRatio	%	0.55	Estimated based on muscle fiber type (relative MHC 1 composition) of subject 1 that was obtained from participation in a previous study [21].
m2Efficency		0.5	Estimated
InternalWork	mol ATP per h	2	Estimated
type1tresh	mol ATP per h	9	Estimated
type2target		0.55	Estimated
peripheralFA	mol fatty acid per h	0	Currently not in use
peripheralLactateCapacity	mol lactate per h	0	This parameter was investigated in Figure S7
HMR_6914	mmol gdw^{-1} h^{-1}	1.25	Complex IV max, Calculated from proteomics Figure 2c.
HMR_6921	mmol gdw^{-1} h^{-1}	0.88	Complex I max, calculated from proteomics Figure 2c.
HMR_6911	mmol gdw^{-1} h^{-1}	0.06	ETF max, fitted for each subject, see Figure S4
scalingFactor		2.96	Fitted for each subject, see Figure S4
Maintainance	mol ATP per h	5.5	Fitted for each subject, see Figure S4
lactateBuffering	mol lactate per h	1.3	Manually fitted
vO2max	mol O2 per h	11.91	Measured for each subject

Supplementary Discussion

Differential expression of metabolic enzymes is prevalent under paraphysiological conditions. There is often an inverse relationship between enzymes from OXPHOS and glycolysis in muscle in paraphysiological conditions[22], consistent with a reallocation of the enzyme pool. Studies have shown that chronic hypoxia is accompanied by increased levels of OXPHOS proteins including complex I at the expense of glycolytic enzymes and lactate dehydrogenase, whilst the opposite occurs as the acute adaptation to hypoxia[22]. A different pattern of protein reallocation is seen in studies of hypoxia in skeletal muscle of mouse[23]; there the activity of Complex I is doubled after 2 days of exposure but returns to baseline after 10 days, potentially due to adaptations that restore oxygen availability on the cellular level. Also other forms of metabolic adaptations have been proposed, e.g. rewiring of the TCA cycle using glutamine as carbon source[23]. Patients suffering from complex I deficiency have a lower VO_2max compared with healthy control[24], consistent with the assumption that complex I is required to support complex I bypass. For these patients, a metabolic bypass of complex I, using a membrane-permeable form of succinate, has been proposed as treatment, and shown effective in complex I deficient blood cells[25]. In muscular dystrophy the levels of OXPHOS proteins are increased[22], but the oxidative capacity per gram of muscle is decreased and the emission of H_2O_2 by complex I is elevated[26]. A reduced ability to bypass complex I would be consistent with the impaired oxidative capacity despite increased levels of OXPHOS proteins, as well as with increased reliance on flux through the H_2O_2 producing complex I. It could also result in elevated membrane potential, which has been liked to increased production of H_2O_2[27].

Supplementary Methods

Manual curation of the muscle GEM

The genome scale model HMR 2.0 [28] contains 3765 genes associated with over 8000 reactions and over 3000 unique metabolites and was used as a scaffold. Reactions that had previously [29] been identified as non-expressed in proteomics and transcriptomics data of human myocytes were removed from the model. A number of different manual curation steps were also performed as specified below resulting in a manually curated muscle GEM with 2951 genes, barely 7000 reactions and 2760 unique metabolites.

ATP synthesis is at the core of muscle metabolism. The stoichiometry of ATP synthase (HMR_6916) was adjusted from 4 H^+ to 3 H^+ per ATP in accordance with literature estimates, which gives a P/O ratio close to 2.5 when oxidizing NADH [30]. A stoichiometry of 4 H^+ is commonly used to account for the proton expenditure of pumping phosphate back in to the mitochondria, but this reaction is already present in the genome-scale model (HMR_5043). To be able to synthesize ATP the uptake of different carbon sources was enabled. A glycogen consumption reaction was added (EC 2.4.1.1). The reaction described the consumption of one glycosyl subunit and one phosphate to produce one glucose-1-phosphate. Uptake reactions of free fatty acids from the extracellular space to the cytosol were also added.

The ability to handle lactate appropriately is important for the model's performance. The idea of an intercellular lactate shuttle between the cytosol and mitochondria is controversial as the experimental data is conflicting and the thermodynamic feasibility has been questioned [31]. To prevent this opportunity in the model, the mitochondrial lactate dehydrogenase reaction (HMR_4280) was blocked. A hypothetical lactate dehydrogenase reaction using ferricytochrome as electron acceptor (HMR_8514) was also blocked due to lack of experimental evidence. It is listed as a probable D-lactate dehydrogenase based on similarities with yeast in Uniprot [32]. Finally a proton-independent lactate transporter was added. The MCT transporter is electron-neutral, one monocarboxylate anion is transported together with a proton [33], since HMR 2.0 does not model charge (e.g. for lactate), the default formulation (HMR_5998) that relied on co-transport of protons imposed an artificial proton cost on lactate secretion.

To allow the transition between a reduced GEM and the muscle GEM, reactions involved in metabolism were curated. The glycerol phosphate shuttle was curated, the reaction from sn-glycerol-3-phosphate to DHAP (HMR_0483) was allowed to proceed in the canonical direction[34]. Alternative formulations using FAD (HMR_0482) were blocked since FAD is an enzyme-bound cofactor in the reaction. The same applied for the succinate to fumarate reaction (HMR_8743). For several fatty acid reactions (e.g. HMR_3211) the FAD was misannotated as ubiquinone, which bypassed the requirement of the ETF reaction (HMR_6911). These reactions were blocked together with a similar reaction involving proline (HMR_3838). To prevent other oxygen consuming reactions than complex IV (HMR_6914), reactions consuming oxygen in the cytosol were removed, excluding reactions involved in transport and fatty acid oxidation. Additionally, the reactions involved in NADPH and NADH were curated so that NADPH reactions were only involved in anabolism and NADH reactions in catabolism.

Reduction of the muscle GEM to a small-scale model

The muscle GEM was reduced to a small-scale model by removing reactions that did not carry flux when maximizing ATP synthesis under various conditions. Some additional restrictions were imposed to comply with the canonical reactions involved in ATP synthesis. All subcellular compartments were removed apart from the extra cellular compartment, the cytosol and the mitochondria. All transport between mitochondria and cytosol was blocked apart from the malate-aspartate shunt (HMR_3949, HMR_3825 and HMR_4852), uptake of pyruvate (HMR_4926) exchange of CO_2, O_2 and H_2O (HMR_4922, HMR_4898 and HMR_4888) as well as exchange of phosphate, ATP and protons (HMR_5043, HMR_6328 and HMR_7638). Water was allowed to freely exchange over the boundary (HMR_9047) but the uptake was blocked for all molecules apart from octanoic acid (HMR_9813), glycogen (HMR_9729) and O_2 (HMR_9048) and the production was blocked for all molecules apart from CO_2 (HMR_9058) and lactate (HMR_9135). The following simulations were performed in the constrained GEM, and reactions that did not carry flux were removed. Maximizing ATP in the presence of fatty acids and glycogen and after blocking complex I (HMR_6921), glycerol-phosphate shunt (HMR_0483), ATP synthase (HMR_6916) and oxygen uptake (HMR_9048) respectively. The resulting model had 95 reactions (out of which 66 were associated with EC codes) and 76 unique metabolites.

Construction of the multi tissue model

The stochiometric matrixes of three identical muscle models (m1, m2 and m3) were concatenated to a joint matrix by adding a compartment for exchange of metabolites between the models and exchange over the boundary (Figure 8). A few auxiliary reactions were added to the joint model, an ATP hydrolysis reaction in each compartment corresponding to the work performed by this sub model, for m3 this was coined maintenance. A reaction for the joint ATP synthesis by m1 and m2, a reaction corresponding to ventilation, exchanging O_2 for CO_2, and finally a reaction for the buffering of lactate by bicarbonate system (lactate => CO2 + H2O).

Pseudo code, Pareto plot (Figure1B)

Data:

S = stoichiometric matrix (mets × rxns) for a reduced model of intermediary metabolism
SA = specific activity vector (rxns) with data for each enzyme (μmol mg^{-1} min^{-1})

Parameters:

P = protein constraint g per gdw, an arbitrary number, $0<P\leq 1$

Functions:

[v f]=linprog(problem), solves a linear programming problem and outputs a vector v with the solution and a scalar f with the value of the objective function.

Output:

The estimated mass required for different flux distributions.

Algorithm:

1. Calculate a vector of weights w in the unit g $mmol^{-1}$ h^{-1} from the specific activity values and assuming half saturation.

$$w_i = \frac{1}{SA \cdot 60 \cdot 0.5} \tag{1}$$

2. Calculate the maximum ATP synthesis capacity under the specific activity constraint using linprog, where c_{ATP} is an all zero vector apart from a 1 at the position of the ATP hydrolysis reaction.

$$[v\ ATPmax] = linprog\begin{pmatrix} maximize\ c_{ATP}^{T} \times v \\ Sv = 0 \\ \sum w_i |v|_i \leq P \end{pmatrix} \tag{2}$$

3. For multiple ATP hydrolysis rates (r) on the interval [0 ATPmax], calculate the minimal substrate uptake, where c_{sub} is an all zero vector apart from a 6 at the position of the glycogen uptake reaction and 16 at the position of palmitate uptake.

$$[v\ substrate\ uptake] = linprog\begin{pmatrix} minimize\ c_{sub}^{T} \times v \\ Sv = 0 \\ v_{[ATP\ hydrolysis]} = r \\ \sum w_i |v|_i \leq P \end{pmatrix} \tag{3}$$

4. Plot X vs Y, where

$$X = \frac{\text{ATP hydrolysis rates}}{glycogen\ uptake} \quad , \quad Y = \frac{\text{ATP hydrolysis rates}}{P} \tag{4}$$

For the extreme states, identify their flux vector v, and estimate the protein requirement as

$$P = \sum w_i |v|_i \tag{5}$$

Pseudo code, fiber simulation (Figure 2B)

Data:

S = stoichiometric matrix (mets × rxns) for a reduced model of intermediary metabolism
SA = specific activity vector (rxns) with data for each enzyme (μmol mg^{-1} min^{-1})
P = proteomics concentrations vector (rxns) for each enzyme (g per gdw)

Output:

The optimal flux distributions for different ATP hydrolysis rates assuming substrate minimization.

Functions:

[v f]=linprog(problem), solves a linear programming problem and outputs a vector v with the solution and a value f with the value of the objective function.

Algorithm:

1. Calculate the vmaxes (mmol per h) of the reactions assuming half saturation

$$vmax = SA \cdot P \cdot 60 \cdot 0.5 \tag{6}$$

2. Calculate the maximum ATP production capacity under the vmax constraint using linprog, where c_{ATP} is an all zero vector apart from a 1 at the position of the ATP hydrolysis reaction.

$$[v\ ATPmax] = linprog\begin{pmatrix} maximize\ c_{ATP}^{T} \times v \\ Sv = 0 \\ v \leq vmax \end{pmatrix} \tag{7}$$

3. For multiple ATP hydrolysis rates (r) on the interval [0 ATPmax], calculate the minimal substrate uptake, where c_{sub} is an all zero vector apart from a 6 at the position of the glycogen uptake reaction and 8 at the position of octanoic acid uptake.

$$[v\ glycogen\, uptake] = linprog\begin{pmatrix} minimize\ c_{glc}^{T} \times v \\ Sv = 0 \\ v_{[ATP\ hydrolysis]} = r \\ v \leq vmax \end{pmatrix} \tag{8}$$

4. Plot fluxes of interest, e.g. X vs Y where

$$X = \text{ATP hydrolysis rates} \quad , \quad Y = v_{[O2\ uptake]} \tag{9}$$

For the comparison of flux and flux capacity (Figure 2C), for all i let Y_i be the maximum value of v_i amongst the iterations in step 3. X = vmax. Plot log of X vs log of Y.

Pseudo code, maximum oxygen uptake simulation (Figure 2D)

Data:

S = stoichiometric matrix (mets × rxns) for a reduced model of intermediary metabolism
SA = specific activity vector (rxns) with data for each enzyme (µmol mg^{-1} min^{-1})
P = proteomics concentrations vector (rxns) for each enzyme (g per gdw)

Output:

The oxygen uptake rate at the maximum attainable ATP synthesis rate.

Functions:

[v f]=linprog(problem), solves a linear programming problem and outputs a vector v with the solution and a value f with the value of the objective function.

Algorithm:

1. Calculate the vmaxes (mmol per h) of the reactions assuming half saturation

$$vmax = SA \cdot P \cdot 60 \cdot 0.5 \tag{10}$$

2. Maximize ATP synthesis capacity under the vmax constraint using linprog under different nutrient conditions, where c_{ATP} is an all zero vector apart from a 1 at the position of the ATP hydrolysis reaction.

$$[v\ ATPmax] = linprog\begin{pmatrix} maximize\ c_{ATP}^T \times v \\ Sv = 0 \\ v \leq vmax \end{pmatrix} \tag{11}$$

   for the following conditions:

   - **OC:** unconstrained uptake of octanoic acid.
   - **GM:** unconstrained flux through the added reaction aspartate -> glutamate ($v_{asp\text{-}glu}$), that allows uptake of glutamate in exchange for aspartate.
   - **GMS:** as GM with the addition of unconstrained flux through the added reaction fumarate -> succinate ($v_{fum\text{-}suc}$) that allows uptake of succinate in exchange for fumarate.
   - **PGMSOC:** as GMS with addition of unconstrained uptake of octanoic acid.
   - **GMSu:** as GMS, with addition of unconstrained flux through the proton pumping OXPHOS reactions, complex I (HMR_6921), complex III (HMR_6918), complex IV (HMR_6914) and complex V (HMR_6916).

3. Repeat step 2 without complex I bypass, i.e. with ub $v_{[GLY\text{-}PHOS]}$=0.

Pseudo code, multi-tissue model (Figure 4)

Data:

S = stoichiometric matrix (mets × rxns) for a connected model with 3 muscle submodels.

Estimated Parameters:

dwMuscle = muscle mass (kg dry weight)
muscleRatio = fraction of type 1 muscle type
m2Efficency = enzyme capacity ratio (m2/m1).
InternalWork = ATP expenditure required to overcome internal resistance at 0 W.
type1tresh = ATP synthesis rate before m2 activation.
type2target = target for m2 share of total ATP.
HMR_6914 = complex IV max, calculated from proteomics data (mmol gdw^{-1} h^{-1})
HMR_6921 = complex I max, calculated from proteomics data (mmol gdw^{-1} h^{-1})
lactateBuffering = maximum rate of lactate buffering in blood (mol per h)

Fitted Parameters:

HMR_6911= ETF max (mmol gdw^{-1} h^{-1})
scalingFactor = a scaling factor for the differences in enzyme concentrations amongst subjects.
maintainance = basal ATP expenditure for maintenance (mol per h)

Measured parameters:

VO2max = maximum oxidative capacity (mol per h)

Output:

Flux distributions including exchange fluxes.

Functions:

[v f]=linprog(problem), solves a linear programming problem and outputs a vector v with the solution and a value f with the value of the objective function.

Notation:

We introduce the following notation for convenience, $v_{X,Y}$ is an element of the vector v corresponding to the reaction Y in the sub-model X. The m1 sub-model corresponds to oxidative type I muscle fibers, m2 to glycolytic type II fibers, b corresponds to the blood compartment, and s corresponds to the boundary.

Algorithm:

1. Modify the stoichiometry of the joint ATP synthesis reaction from m1 and m2 (jointATP) to *type2target* for m2 and (*1-type2target*) for m1.

2. Set lower bounds on ATP synthesis for maintenance in m3 and internal work in m1 in the lower bounds vector (lb).

$$lb_{m3,ATPmaintainance} = maintainance$$
$$lb_{m1,ATPmaintainance} = internalWork$$

3. Set upper bounds (ub) on the lactate buffering capacity and oxygen uptake capacity.

$$ub_{b,buffering} = lactateBuffering$$
$$ub_{s,O2In} = VO2max$$

4. Set upper bounds on the capacity of complex IV (HMR_6914), complex I (HMR_6921) and on ETF (HMR_6911) in the upper bounds vector (ub). The capacity constraints depend on muscle-type dependent scaling factors (α):

$$\alpha_{m1} = dwMuscle \cdot scalingFactor \ \cdot muscleRatio$$
$$\alpha_{m2} = dwMuscle \cdot scalingFactor \ \cdot (1 - muscleRatio) \cdot m2Efficency$$
$$ub_{m1,HMR_{6914}} = \alpha_{m1} \cdot HMR_{6914}$$
$$ub_{m2,HMR_{6914}} = \alpha_{m2} \cdot HMR_{6914}$$
$$ub_{m1,HMR_{6921}} = \alpha_{m1} \cdot HMR_{6921}$$
$$ub_{m2,HMR_{6921}} = \alpha_{m2} \cdot HMR_{6921}$$
$$ub_{m1,HMR_{6911}} = \alpha_{m1} \cdot HMR_{6911}$$
$$ub_{m2,HMR_{6911}} = \alpha_{m2} \cdot HMR_{6911}$$

5. Set upper bounds (ub) on the ATP synthesis using only type 1 muscle

$$ub_{m1,ATPtoWork} = type1tresh$$

6. Calculate the maximum ATP synthesis rate under the capacity constraints using linprog, where c_{ATP} is an all zero vector apart from a 1 at the position of the m1 ATPtoWork reaction and the joint ATP reaction.

$$[v\ ATPmax] = linprog\begin{pmatrix} maximize\ c_{ATP}^T \times v \\ Sv = 0 \\ lb \leq v \leq ub \end{pmatrix} \tag{12}$$

7. Construct an objective function (the vector c_{obj}) for minimization of oxygen and substrate utilization and to disfavor lactate synthesis and ATP synthesis by m2.

$$c_{s,O2In} = -1$$
$$c_{glycogenIn} = -1$$
$$c_{m1,lactateIn} = -0.9$$
$$c_{b,buffering} = -3$$
$$c_{m1,ATPtoWork} = 0.01$$

8. For multiple ATP hydrolysis rates on the interval [0 ATPmax], calculate the optimal fluxes given the objective function c_{obj}.

$$[v\ arbitaryValue] = linprog\begin{pmatrix} minimize\ c_{obj}^T \times v \\ Sv = 0 \\ lb \leq v \leq ub \end{pmatrix} \tag{13}$$

9. Plot relevant fluxes, e.g oxygen expenditure against watt (using conversion-factor from ATP).

Pseudo code, steady-state watt max simulation (Figure 5a)

Data:

See connected model.

Fitted Parameters:

See connected model.

Output:

Maximum ATP synthesis capacity under different conditions.

Functions:

See connected model.

Algorithm:

1. Follow steps 1-5 for the multi-tissue model.

2. Constrain the uptake to the specified substrates (glycogen, glucose, fat) and set arbitrary large parameter values to FAFactor and vO2max to study these conditions.

3. Calculate the maximum ATP synthesis rate using linprog, where c_{ATP} is an all zero vector apart from a 1 at the position of the m1 ATPtoWork reaction and the joint ATP reaction.

$$[v\ ATPmax] = linprog\begin{pmatrix} maximize\ c_{ATP}^T \times v \\ Sv = 0 \\ lb \leq v \leq ub \end{pmatrix} \tag{14}$$

4. Compare the ATP synthesis capacity for the different substrates.

Pseudo code, watt max simulation with substrate depletion (Figure 5b)

Data:

See connected model.
GlycogenPoolSize = the pool sizes of muscle glycogen (mol).
GlucosePoolSize = the pool sizes of liver glucose (mol).
LactatePoolSize = the pool of lactate based on the maximum lactate concentration in blood (mol).

Fitted Parameters:

See connected model.

Output:

Maximum ATP synthesis capacity for different exercise durations.

Functions:

See connected model.

Algorithm:

1. Follow steps 1-5 in multi tissue model.

2. To avoid ambiguity of the solutions set up a secondary objective function to disfavor lactate production and ATP synthesis by m2.

$$
\begin{aligned}
c_{s,O2In} &= -1 \\
c_{glycogenIn} &= -1 \\
c_{glucoseIn} &= -1 \\
c_{m1,lactateIn} &= -0.9 \\
c_{b,buffering} &= -3 \\
c_{m1,ATPtoWork} &= 0.01
\end{aligned}
$$

3. For multiple durations (d) on the interval [4 130] minutes. Constrain the uptake of nutrients based on the pool size and duration and calculate the maximum ATP production capacity under the oxidative capacity constraints using linprog. To avoid ambiguity of the solutions a second optimization is performed with the vector c_{obj}.

$$[v\ ATPmax] = linprog\left(\begin{array}{c} maximize\ c_{ATP}^{T} \times v \\ Sv = 0 \\ lb \leq v \leq ub \\ v_{LactateOut} = \dfrac{LactatePoolSize}{d/60} \\ v_{m1,glcIn} = \dfrac{GlycogenPoolSize \cdot muscleRatio}{d/60} \\ v_{m2,glcIn} = \dfrac{GlycogenPoolSize \cdot (1 - muscleRatio)}{d/60} \\ v_{glcIn} = \dfrac{GlucosePoolSize}{d/60} \end{array}\right) \quad (15)$$

$$[v\ arbitaryValue] = linprog\left(\begin{array}{c} minimize\ c_{obj}^{T} \times v \\ v_{ATPOut} = ATPmax \\ and\ as\ above \end{array}\right) \quad (16)$$

4. Compare the ATP synthesis capacity for the different durations.

Pseudo code, slow component simulation (Supplementary Figure 7)

Data:

lactateReservoir = total volume in which lactate may be stored (l)
lactateFlux= the rate at which lactate is produced
maxLactateFlux = the maximum flux of lactate at watt max
basalConcentration = the lactate concentration at rest
K = Michaelis–Menten kinetic parameters for lactate dehydrogenase.
lactateEfficency = the relative efficiency of lactate, estimated as 86% based on resting oxygen expenditure of 1350 vs 1570 ml per min in the presence and absence of lactate.

Functions:W

[t y]=ODE(problem, init): solves an ordinary differential equation problem (problem) with some boundary conditions (init).
v=MM(S,K): the rate (v) of the Michaelis–Menten equation for a concentration (S) and kinetic parameters (K).
y=vO2(x): convert the oxygen flux in mol per h to ml per min

Output:

The predicted time dynamics of lactate and oxygen at different work rates.

Algorithm:

1. Calculate basal lactate flux

$$lactateFlux = MM(\text{basalConcentration}, \text{K}) \tag{17}$$

2. Solve an ODE problem for the accumulation of lactate under this condition.

$$[v\ ATPmax] = ODE\left(\frac{dy}{dt} = basalFlux + lactateFlux - MM\left(\frac{y}{lactateReservoir}, K\right)\right) \tag{18}$$

3. Calculate the expected oxygen consumption from consumption of lactate as

$$oxygenFromLactate = vO2(lactateFlux \cdot 3) \tag{19}$$

4. Calculate oxygen consumption based on empirical formula from literature [2]:

$$oxygenEmpirical = \begin{matrix} t < 9.3 & 1350 \\ t \geq 9.3 & 1350 + 1800 \cdot \left(1 - e^{\frac{-(t-9.3)}{24.9}}\right) \end{matrix} \tag{20}$$

5. Calculate the fraction of oxygen consumption from lactate as:

$$lactateFraction = \begin{matrix} oxygenFromLactate > oxygenEmpirical & = 1 \\ oxygenFromLactate \leq oxygenEmpirical & = \dfrac{oxygenFromLactate}{oxygenEmpirical} \end{matrix} \tag{21}$$

6. Calculate the modified oxygen consumption rate as:

$$oxygenModified = \frac{oxygenEmpirical}{lactateEfficency\ \cdot lactateFraction + 1 \cdot (1 - lactateFraction)} \tag{22}$$

Supplementary References

1. Talaiezadeh, A., Shahriari, A., Tabandeh, M. R., Fathizadeh, P. & Mansouri, S. Kinetic characterization of lactate dehydrogenase in normal and malignant human breast tissues. *Cancer Cell Int.* **15**, 19 (2015).
2. Sahlin, K., Sørensen, J. B., Gladden, L. B., Rossiter, H. B. & Pedersen, P. K. Prior heavy exercise eliminates V̇o(2) slow component and reduces efficiency during submaximal exercise in humans. *J. Physiol.* **564**, 765–773 (2005).
3. Korzeniewski, B. & Rossiter, H. B. Each-step activation of oxidative phosphorylation is necessary to explain muscle metabolic kinetic responses to exercise and recovery in humans. *J. Physiol.* **593**, 5255–5268 (2015).
4. Baccanari, D. P. & Cha, S. Succinate Thiokinase: VI. MULTIPLE INTERCONVERTIBLE FORMS OF THE ENZYME . *J. Biol. Chem.* **248**, 15–24 (1973).
5. Pawelczyk, T. & Angielski, S. Cooperation of Ca2+ and pH in regulation of the activity of the 2-oxoglutarate dehydrogenase complex and its components from bovine kidney cortex. *Acta Biochim. Pol.* **31**, 289–305 (1984).
6. Higuchi, H. & Goldman, Y. E. Sliding distance per ATP molecule hydrolyzed by myosin heads during isotonic shortening of skinned muscle fibers. *Biophys. J.* **69**, 1491–1507 (1995).
7. Bateman, A. *et al.* UniProt: The universal protein knowledgebase. *Nucleic Acids Res.* **45**, D158–D169 (2017).
8. Sinjorgo, K. M. C. *et al.* Human cytochrome c oxidase isoenzymes from heart and skeletal muscle; purification and properties. *Biochim. Biophys. Acta - Bioenerg.* **890**, 144–150 (1987).
9. Choksi, K. B., Boylston, W. H., Rabek, J. P., Widger, W. R. & Papaconstantinou, J. Oxidatively damaged proteins of heart mitochondrial electron transport complexes. *Biochim. Biophys. Acta - Mol. Basis Dis.* **1688**, 95–101 (2004).
10. Matsuno-yagi, A. & Hatefi, Y. Estimation of the Turnover Number of Bovine Heart FoF1 Complexes for ATP Synthesis ? *Biochemistry* **27**, 335–340 (1988).
11. Schomburg, I. *et al.* BRENDA in 2013: integrated reactions, kinetic data, enzyme function data, improved disease classification: new options and contents in BRENDA. *Nucleic Acids Res.* **41**, D764-72 (2013).
12. Letts, J. A., Degliesposti, G., Fiedorczuk, K., Skehel, M. & Sazanov, L. A. Purification of Ovine Respiratory Complex I Results in a Highly Active and Stable Preparation. *J. Biol. Chem.* **291**, 24657–24675 (2016).
13. King, M. S., Sharpley, M. S. & Hirst, J. Reduction of Hydrophilic Ubiquinones by the Flavin in Mitochondrial NADH:Ubiquinone Oxidoreductase (Complex I) and Production of Reactive Oxygen Species. *Biochemistry* **48**, 2053–2062 (2009).
14. Grivennikova, V. G., Kapustin, A. N. & Vinogradov, A. D. Catalytic Activity of NADH-ubiquinone Oxidoreductase (Complex I) in Intact Mitochondria: EVIDENCE FOR THE SLOW ACTIVE/INACTIVE TRANSITION . *J. Biol. Chem.* **276**, 9038–9044 (2001).
15. Galkin, A. & Brandt, U. Superoxide Radical Formation by Pure Complex I (NADH:Ubiquinone Oxidoreductase) from Yarrowia lipolytica. *J. Biol. Chem.* **280**, 30129–30135 (2005).
16. Angerer, H. *et al.* The LYR protein subunit NB4M/NDUFA6 of mitochondrial complex I anchors an acyl carrier protein and is essential for catalytic activity. *Proc. Natl. Acad. Sci.* **111**, 5207 LP – 5212 (2014).
17. Vinogradov, A. D. & Grivennikova, V. G. Oxidation of NADH and ROS production by respiratory complex I. *Biochim. Biophys. Acta - Bioenerg.* **1857**, 863–871 (2016).
18. Andreani, A. *et al.* Effects of new ubiquinone-imidazo[2,1-b]thiazoles on mitochondrial complex I (NADH-ubiquinone reductase) and on mitochondrial permeability transition pore. *Bioorg. Med. Chem.* **12**, 5525–5532 (2004).
19. Vinogradov, A. D. Catalytic properties of the mitochondrial NADH–ubiquinone oxidoreductase (Complex I) and the pseudo-reversible active/inactive enzyme transition. *Biochim. Biophys. Acta - Bioenerg.* **1364**, 169–185 (1998).
20. Vinogradov, a D. & Grivennikova, V. G. The mitochondrial complex I: progress in understanding of catalytic properties. *IUBMB Life* **52**, 129–34 (2001).
21. Niklas, P., Li, W., Jens, W., Michail, T. & Kent, S. Mitochondrial gene expression in elite cyclists: Effects of high-intensity interval exercise. *Eur. J. Appl. Physiol.* **110**, 597–606 (2010).
22. Gelfi, C., Vasso, M. & Cerretelli, P. Diversity of human skeletal muscle in health and disease: Contribution of proteomics. *J. Proteomics* **74**, 774–795 (2011).
23. Capitanio, D. *et al.* TCA cycle rewiring fosters metabolic adaptation to oxygen restriction in skeletal muscle from rodents and humans. *Sci. Rep.* **7**, 9723 (2017).
24. Wu, F., Jeneson, J. A. L. & Beard, D. A. Oxidative ATP synthesis in skeletal muscle is controlled by substrate feedback. *Am. J. Physiol. Physiol.* **292**, C115–C124 (2007).
25. Ehinger, J. K. *et al.* Cell-permeable succinate prodrugs bypass mitochondrial complex I deficiency. *Nat. Commun.* **7**, 12317 (2016).
26. Hughes, M. C. *et al.* Early myopathy in Duchenne muscular dystrophy is associated with elevated mitochondrial H2O2 emission during impaired oxidative phosphorylation. *J. Cachexia. Sarcopenia Muscle* **0**, (2019).
27. LAMBERT, A. J. & BRAND, M. D. Superoxide production by NADH:ubiquinone oxidoreductase (complex I) depends on the pH gradient across the mitochondrial inner membrane. *Biochem. J.* **382**, 511 LP – 517 (2004).

28. Mardinoglu, A. *et al.* Genome-scale metabolic modelling of hepatocytes reveals serine deficiency in patients with non-alcoholic fatty liver disease. *Nat. Commun.* **5**, 3083 (2014).
29. Väremo, L. *et al.* Proteome- and Transcriptome-Driven Reconstruction of the Human Myocyte Metabolic Network and Its Use for Identification of Markers for Diabetes. *Cell Rep.* **11**, 921–933 (2015).
30. Hinkle, P. C. P/O ratios of mitochondrial oxidative phosphorylation. *Biochimica et Biophysica Acta - Bioenergetics* **1706**, 1–11 (2005).
31. Gladden, L. B. Lactate metabolism: a new paradigm for the third millennium. *J. Physiol.* **558**, 5–30 (2004).
32. UNIPROT. Activities at the Universal Protein Resource (UniProt). *Nucleic Acids Res.* **42**, D191-8 (2014).
33. Pösö, A. R. Monocarboxylate transporters and lactate metabolism in equine athletes: a review. *Acta Vet. Scand.* **43**, 63–74 (2002).
34. Mráček, T., Drahota, Z. & Houštěk, J. The function and the role of the mitochondrial glycerol-3-phosphate dehydrogenase in mammalian tissues. *Biochim. Biophys. Acta - Bioenerg.* **1827**, 401–410 (2013).
