# Supplementary material for: Complex I is bypassed during high intensity exercise
Source: Nat Commun. 2019 Nov 7;10:5072. doi: 10.1038/s41467-019-12934-8 (PMC6838197; doi:10.1038/s41467-019-12934-8)
Supplement: Supplementary file 4 — Reporting Summary [file 41467_2019_12934_MOESM4_ESM.pdf]

nature research

Corresponding author(s): Jens Nielsen

Reporting Summary

Nature Research wishes to improve the reproducibility of the work that we publish. This form provides structure for consistency and transparency in reporting. For further information on Nature Research policies, see Authors & Referees and the Editorial Policy Checklist.

Statistical parameters

When statistical analyses are reported, confirm that the following items are present in the relevant location (e.g. figure legend, table legend, main text, or Methods section).

n/a	Confirmed	
☐	☒	The exact sample size (n) for each experimental group/condition, given as a discrete number and unit of measurement
☐	☒	An indication of whether measurements were taken from distinct samples or whether the same sample was measured repeatedly
☐	☒	The statistical test(s) used AND whether they are one- or two-sided<br>*Only common tests should be described solely by name; describe more complex techniques in the Methods section.*
☒	☐	A description of all covariates tested
☐	☒	A description of any assumptions or corrections, such as tests of normality and adjustment for multiple comparisons
☐	☒	A full description of the statistics including central tendency (e.g. means) or other basic estimates (e.g. regression coefficient) AND variation (e.g. standard deviation) or associated estimates of uncertainty (e.g. confidence intervals)
☐	☒	For null hypothesis testing, the test statistic (e.g. F, t, r) with confidence intervals, effect sizes, degrees of freedom and P value noted<br>*Give P values as exact values whenever suitable.*
☒	☐	For Bayesian analysis, information on the choice of priors and Markov chain Monte Carlo settings
☒	☐	For hierarchical and complex designs, identification of the appropriate level for tests and full reporting of outcomes
☐	☒	Estimates of effect sizes (e.g. Cohen's d, Pearson's r), indicating how they were calculated
☐	☒	Clearly defined error bars<br>*State explicitly what error bars represent (e.g. SD, SE, CI)*

Our web collection on statistics for biologists may be useful.

Software and code

Policy information about availability of computer code

Data collection	O2 and CO2 measurements, Oxycon Pro (Erich Jaeger GmbH, Hoechberg, Germany)<br>Power measurements, SRM Ergometer (SRM international, Jülich Germany)
Data analysis	Custom code run under Matlab R2018b (The MathWorks, Inc., Natick, Massachusetts, United States)

For manuscripts utilizing custom algorithms or software that are central to the research but not yet described in published literature, software must be made available to editors/reviewers upon request. We strongly encourage code deposition in a community repository (e.g. GitHub). See the Nature Research guidelines for submitting code & software for further information.

Data

Policy information about availability of data

All manuscripts must include a data availability statement. This statement should provide the following information, where applicable:

- Accession codes, unique identifiers, or web links for publicly available datasets
- A list of figures that have associated raw data
- A description of any restrictions on data availability

The watt and gas exchange measurements displayed is available in Supplementary Data 1.

Field-specific reporting

Please select the best fit for your research. If you are not sure, read the appropriate sections before making your selection.

☒ Life sciences ☐ Behavioural & social sciences ☐ Ecological, evolutionary & environmental sciences

For a reference copy of the document with all sections, see nature.com/authors/policies/ReportingSummary-flat.pdf

Life sciences study design

All studies must disclose on these points even when the disclosure is negative.

Sample size	no sample-size calculation was performed, since experimental groups were not used. The number of data points (N=135) were given by the length of the experiment (~40 min), which was selected based on the subjects expected time to exhaustion. The data points correspond to the mean values of non-overlapping 15 second time windows from continuously measured gas exchange.
Data exclusions	O2 and CO2 data measured during transitions (resting to pedaling) that not reflect steady state gas exchange were excluded. All measured data was thoroughly examined and data was excluded based on manual notes from observations during test sessions regarding: transition in power, alternation in cadence, compliance and quality of measurement of gas and ventilation.
Replication	The measurements were repeated at both low and intermediary work rates and were found consistent with the full range measurements.
Randomization	Experimental groups was not relevant to this study as no intervention was used
Blinding	Blinding was not relevant to this study as experimental groups were not used.

Reporting for specific materials, systems and methods

Materials & experimental systems

n/a	Involved in the study	
☒	☐	Unique biological materials
☒	☐	Antibodies
☒	☐	Eukaryotic cell lines
☒	☐	Palaeontology
☒	☐	Animals and other organisms
☐	☒	Human research participants

Methods

n/a	Involved in the study	
☒	☐	ChIP-seq
☒	☐	Flow cytometry
☒	☐	MRI-based neuroimaging

Human research participants

Policy information about studies involving human research participants

Population characteristics	Five male subjects (age 38±5 y, height 181±3 cm, weight 76±11 kg, VO2max 62±11 ml/kg-min-1, Wmax 5.2±1 W/kg)
Recruitment	The subjects were recruited based on experience with the laboratory testing equipment. This biased the recruitment towards well trained subjects. This is not expected to affect the results.
