# Supplementary material for: Complex I is bypassed during high intensity exercise
Source: Nat Commun. 2019 Nov 7;10:5072. doi: 10.1038/s41467-019-12934-8 (PMC6838197; doi:10.1038/s41467-019-12934-8)
Supplement: Supplementary file 5 — Description of Additional Supplementary Files [file 41467_2019_12934_MOESM5_ESM.pdf]

Title: Supplementary Data 1.
Description: Gas exchange data for 5 subjects performing an incremental exercise protocol.

Title: Supplementary Data 2.
Description: Metabolic flux distributions predicted by the computational models.
